# Stress Responses of Small Heat Shock Protein Genes in Lepidoptera Point to Limited Conservation of Function across Phylogeny

**DOI:** 10.1371/journal.pone.0132700

**Published:** 2015-07-21

**Authors:** Bo Zhang, Jincheng Zheng, Yu Peng, Xiaoxia Liu, Ary A. Hoffmann, Chun-Sen Ma

**Affiliations:** 1 Group of Climate Change Biology, State Key Laboratory for Biology of Plant Diseases and Insect Pests, Institute of Plant Protection, Chinese Academy of Agricultural Sciences, Beijing, China; 2 Department of Entomology, China Agricultural University, Yuanmingyuan West Road, Beijing, China; 3 School of BioSciences, The University of Melbourne, Melbourne, Victoria, Australia; Washington State University, UNITED STATES

## Abstract

The small heat shock protein (sHsp) family is thought to play an important role in protein refolding and signal transduction, and thereby protect organisms from stress. However little is known about sHsp function and conservation across phylogenies. In the current study, we provide a comprehensive assessment of small Hsp genes and their stress responses in the oriental fruit moth (OFM), *Grapholita molesta*. Fourteen small heat shock proteins of OFM clustered with related Hsps in other Lepidoptera despite a high level of variability among them, and in contrast to the highly conserved Hsp11.1. The only known lepidopteran sHsp ortholog (Hsp21.3) was consistently unaffected under thermal stress in Lepidoptera where it has been characterized. However the phylogenetic position of the sHsps within the Lepidoptera was not associated with conservation of induction patterns under thermal extremes or diapause. These findings suggest that the sHsps have evolved rapidly to develop new functions within the Lepidoptera.

## Introduction

Heat shock protein (HSP) families have been widely associated with physiological processes and stress resistance. The small heat shock proteins (sHSPs) (12–43 kDa) represent a particularly heterogeneous [1] family of HSPs, in contrast with other HSPs such as HSP70 and HSP90 that are highly conserved [2,3]. Many sHsp genes are upregulated under thermal stress and may protect insects against stressful conditions; for example Hsp26 is thought to protect *Aedes aegypti* larvae and pupae from heat stress [4], while Hsp20 may underlie a tradeoff between thermal protection at the cost of reproductive impairment in *Liriomyza huidobrensis* [5]. The sHsp genes may also play a role in cold tolerance, as suggested by the upregulation of Hsp20 expression in the recovery of *Drosophila melanogaster* from cold injury [6]. Hsp23 is strongly upregulated during pupal diapause in the flesh fly, *Sarcophaga crassipalpis* [7,8], while Hsp20.8 expression has been linked to diapause in *Sesamia nonagrioides* [9]. The sHsps therefore represent an important inducible chaperone family involved in stress responses.

The conservation of sHsps has rarely been considered in insects, in contrast to Hsp70 which shows a high degree of evolutionary conservation both in its sequence and in its induction pattern [10,11]. The functional significance of the sHps in many insect orders including the Lepidoptera remains unclear, despite an increasing amount of sequence information becoming available. Lepidopteran sHsps are thought to have evolved independently from sHsps in other orders, except that one ortholog, Hsp21.4-like protein, has been discovered in insect species from multiple orders [12–14]. The Hsp10 family also appears to be highly conserved across insects, and also in vertebrates where it acts as a co-chaperone with Hsp60 [15]. Some studies have considered sHsp expression patterns in Lepidoptera in an attempt to understand their potential function. Changes in expression patterns suggest that the sHsp genes in the armyworm, *Spodoptera litura*, may have roles in cold responses as well as moth metamorphosis [14,16]. In the codling moth, *Cydia pomonella*, three sHps were upregulated under heat stress and were persistently expressed during development [17], suggesting a role in both processes.

The Oriental fruit moth (OFM), *Grapholita molesta*, one of the world’s most invasive orchard pest insect of stone and pome fruits, causes severe economic loss in global fruit industry [18–20]. It is thought to have originated in China and spread to East Asia, Europe, Africa, Australia, and America during last century [20]. OFM has gained recognition as a global pest of peaches and other fruit crops since its introduction into America [21]. Its generation time depends on temperature and latitude, and varies considerably across regions; for example one or two generations occur in northeastern Asia, compared to six to seven annually in Georgia, USA [22,23]. To overwinter in cold environments, OFM diapause at a late larvae stage, which is induced by low temperature and a short photoperiod [18]. The developmental threshold of OFM ranges from 4°C to 11°C at the lower end, and from 30°C to 35°C at the upper end, suggesting a wide thermal performance breadth which has likely assisted their worldwide expansion [19].

Here we describe the sHsps genes in OFM and their expression patterns across developmental stages, across tissues and in response to heat stress, cold stress and diapause. We use these data to address a number of questions. (1) How do the sHsp and Hsp10 genes of OFM compare to those of other Lepidoptera? Do they fall into specific phylogenetic groupings? (2) Are there unique or common patterns of expression changes in the sHsps when they are under the different stresses or at different temporal/spatial stages? (3) Has the putative functional role of particular sHsps as defined by their patterns of induction been conserved across different lepidopteran species, such that related sHsps group together?

## Materials and Methods

### Insect preparation

We collected OFM larvae from an experimental station in Hebei province (N39°47′, E118°41′), where the pests could be sampled without permission. The field studies did not involve endangered or protected species. OFM were reared on fresh apples in the laboratory over two years at 24°C and 60% relative humidity under a photoperiod of 15: 9 (L: D). Six developmental stages of OFM, 3-day-old eggs, 1^st^ instar larvae, 3^rd^ instar larvae, 5^th^ instar larvae, pupae, and adults (equal numbers of males and females), were collected and frozen in liquid nitrogen before being stored at -80°C for further RNA extraction. The 5th instar larvae were dissected on ice, and each tissue including head, thorax, abdomen, fat body, and midgut were kept in RNAlater (Qiagen, Hilden, Germany) at -80°C.

To characterize expression patterns of Hsps in adults under thermal extremes, high and low temperatures were initially defined based on pilot experiments; these were the most severe conditions to which recently-eclosed adults could be exposed in the absence of mortality. Moths that had eclosed within 24 hours were therefore kept at -5°C or 40°C for 1 and 2 hours, and then left to recover at 24°C for 2 hours before being frozen in liquid nitrogen. Controls were treated identically but were not stressed. The thermal treatment is based on Drosophila Hsp studies [6,24] and a pilot experiment, in which different recovery and stress periods were considered. Those sHsps that were responsive under 2 h were also highly responsive under shorter or longer exposure times, while non-responsive sHsps failed to be expressed under all conditions tested. For the diapause treatment, diapause was induced by rearing moths at same temperature of 24°C under a photoperiod of 12 hours [25]. Each treatment consisted of five biological replicates, with 14–20 individuals (7–10 per sex) per replicate.

### RNA extraction and cDNA synthesis

Total RNA was isolated with an RNeasy Mini kit (Qiagen, Valencia, CA, USA) and digested with DNase I (Invitrogen) following supplier’s instructions. The concentration of RNA was measured using a NanoDrop spectrophotometer (ND-1000, Thermo Fisher Scientific, Wilmington, DE). 2 μg of total RNA was used as a template in first strand cDNA synthesis using M-MLV reverse transcriptase following the manufacturer’s instruction with modified oligo (dT) (Promega, Madison, WI, USA). The final 25 μl volume cDNA templates were used for Real-time quantitative PCR of gene expression. The single strand cDNA from the whole body of OFM fifth instar larvae serving as the RACE template was synthesized by 1 μg of total RNA following instructions in the SMART RACE cDNA Amplification Kit (Clontech, Mountain View, CA, USA).

### Clone of full length OFM sHsps

The conserved α-crystalline domain of lepidopteran sHsps was used to design the degenerated primers for full length sHsps in OFM. 5’- and 3’ RACE was done separately by universal primers and gene specific primers with the following cycles: five cycles consisting of 94°C for 5 s, 72°C for 3 min, then five cycles consisting of 94°C for 5 s, 70°C for 10s and 72°C for 3 min, and then 25 cycles consisting of 94°C for 5 s, 68°C for 10 s and 72°C for 180 s. A final extension was performed under 72°C for 10 min. The PCR products were visualized by electrophoresis in a 1.5% agarose gel and purified using an ABgene Ultra PCR Clean-Up Kit (Thermo Scientific) before they were cloned into a pGEM-Teasy vector (Promega, Madison, WI, USA). The products were sequenced on a 3730xl DNA Analyser (Applied Biosystems).

### Real-time quantitative PCR (RT-qPCR)

The sHSP RT qPCR primers were designed based on the full length cDNA transcripts (S1 Table). All cDNA templates were reversely transcribed from 2 μg total RNA in the final 25 μl volume, and 1 μl was used in the RT-qPCR reaction. β-actin and GAPDH were both chosen as the reference genes for normalizing the mRNA expression level, however data were also normalized without using reference genes through the Norma-gene normalization algorithm [26] which involves normalization based on the experimental data rather than reference genes to avoid the issue of error in reference gene expression, and the assumption that the reference genes are not influenced by the different conditions tested. The sHsp transcript standards were produced by cloning the PCR products into plasmids. Seven 10-fold serial dilutions of stock plasmid served as quantitative standards to estimate the relative expression of each sHsp gene transcript. RT-qPCR was conducted in a 20 μl volume comprising of 10 μl 2×SYBR Green PCR Master Mix (Takara, Dalian, China), 1μl cDNA template/plasmid, 1μl RT-qPCR primers (10mM working solution), and 8 μl dd H_2_O. A Stratagene Mx3000P thermal cycler was used. The PCR cycling parameters were as follows: initial denaturation of 10 s at 95°C, and 40 cycles of 5 s at 95°C, 20 s at 55°C, and 20 s at 72°C. Each experimental group included five biological replicates, each of which contained three technical repeats.

Differences between treatments for normalized expression were compared either by t-test (for comparison of two means), or by one-way analysis of variance (ANOVA) followed by a Tukey B test for posthoc comparisons among means, run with SPSS 18.0 software. Treatment differences were considered significant at p < 0.01. Values are denoted as means ± SE (standard error). Unsupervised hierarchical clustering was performed with Cluster v3.0 software using uncentered Pearson correlations and complete linkage, and run with Java TreeView software [27,28].

### Phylogenetic analysis

The NCBI BLAST tool was used to detect sHsps and ensure the fragments sequenced involved sHsp conserved domains. The open reading frames (ORFs), translations, and predicted secondary structure of sHsps were obtained online (http://www.bioinformatics.org/sms2). The molecular weights of sHsps were also estimated by an online tool (http://web.expasy.org/protparam). For comparisons of sHsps across diverse groups, the sHsp gene sequences derived from other insect species were downloaded from GenBank, (S2 Table) and aligned in MEGA6 [29]. All phylogenetic analyses were run using Bayesian Inference (BI), Maximum Likelihood (ML) and Neighbor Joining (NJ) methods. BI analysis was performed using MrBayes, v 3.1.2 [30]. Two sets of four chains were allowed to run simultaneously for 1,000,000 generations. Each set was sampled every 100 generations with a burn-in of 25%. Stationarity was considered to have been reached when the average standard deviation of split frequencies was less than 0.01. Bayesian posterior probabilities (BPP) were estimated based on a 50% majority rule consensus tree of the remaining trees. ML analysis was conducted using an online program (http://www.atgc-montpellier.fr/phyml/) with a GTR substitution model. Bootstrap values were obtained for the nodes based on 1000 replicates. NJ trees were built in MEGA5 based on p-distance and bootstrap values for nodes were based on 1000 replicates.

### Phylogenetic reconstruction and signal detection

To examine the possible evolutionary conservation of sHsp functions in response to temperature and diapause, the expression traits were analyzed to trace phylogenetic signal based on a tree generated through BI. We collected data on temperature responses of lepidopteran species from published references, and characterized sHsp expression into three categories (upregulated, no change, or unknown) under the three conditions considered (heat, cold and diapause). Downregulated patterns were not considered because these were absent from our study and the published literature. We then evaluated the number of evolutionary steps between genes with similar functions (same pattern of expression under a condition) based on parsimony reconstruction, and compared these with the number of steps expected by chance under a null model. Expression characteristics were reshuffled 1000 times across the tips of the phylogeny to obtain confidence intervals and test for significance under p = 0.01. These comparisons were run in Mesquite 1.12 [31].

## Results

### Cloning and characterization of GmHsps

The full-length Hsps were obtained by using 5’ and 3’ rapid amplification of cDNA ends from the cDNA library of the whole body of OFM. Fourteen GmHsp genes were sequenced and deposited in GenBank (KP843895- KP843908). Based on their predicted molecular weight, they were named as GmHsp11.1, 18.9, 19.6, 19.8a, 19.8b, 19.9, 20.4, 21.3, 21.4, 21.7, 22.1, 22.5, 24.8, and 31.8. The ORFs spanned between 312 and 849 bp, coding from 103 to 282 amino acid proteins.

**Fig 1:**
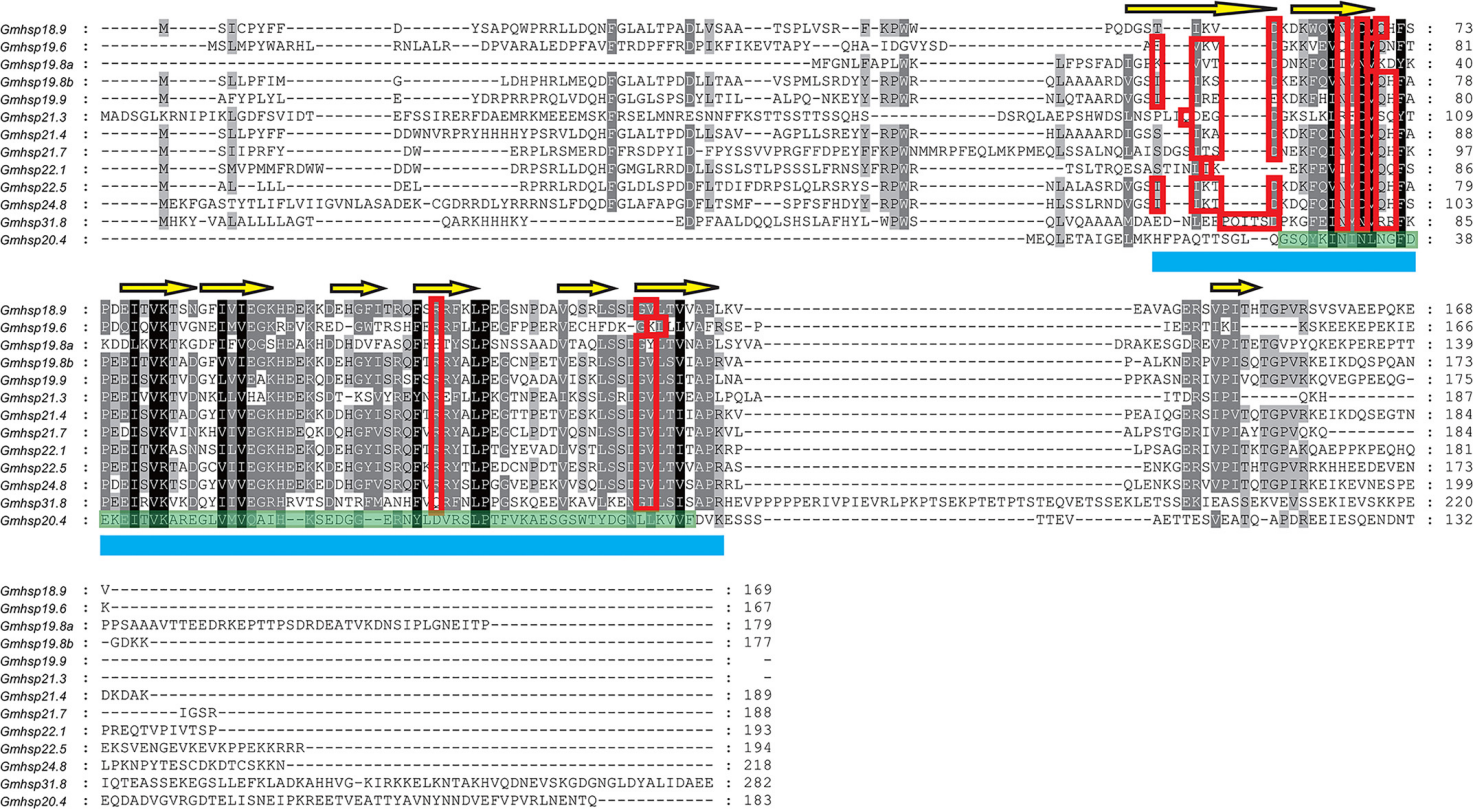
13 sHsp gene alignment of *G*. *molesta*. The predicted secondary structure showing the α-crystallin domain (ACD) is underlined by a blue solid line. Yellow arrows indicate β - strands. Red boxes indicate putative dimer interfaces. The green highlighted fragment is the ACD p23-like superfamily.

**Fig 2:**
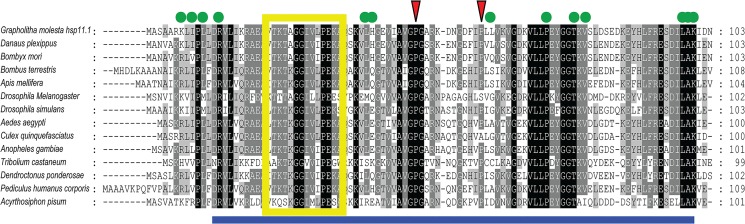
Alignment of Hsp11.1 of *G*. *molesta* with thirteen other insect species. The predicted secondary structure of the Cpn10 superfamily is underlined by a blue solid line. Red arrows show two roof hairpins. Yellow boxes indicate a mobile loop, and green dots indicate 13 putative oligomerisation interfaces.

GmHsps were classified into two families, 13 of them being sHsps and one being Hsp10 (also named as chaperonin 10, Cpn10) (Figs 1 and 2). A conserved α-crystalline domain comprising about 100 amino acids was detected in all OFM sHsps, containing 9 β-sheet sandwich structures numbered β2 to β10 (Fig 1). The typical 12 putative dimer interfaces were found to have highly conserved sites of the metazoan α-crystalline domain (ACD), while GmHsp22.1 only presented eight putative dimer interfaces. While GmHsp20.4 was classified as a sHsp, no dimer interface was found, as its conserved domain was ACD sHsp p23-like. Due to the high divergence of the sHsp sequences, genetic distance comparisons were based on the 105 bp conserved ACD characterized by NCBI. GmHsp20.4 showed higher average genetic distances based on the number of amino acid substitutions per site between sequences, ranging from 1.881 substitutions with GmHsp24.8 to 2.708 with GmHsp19.8a. While C and N terminals showed significant differentiation between the full-length gene of GmHsp21.4 and GmHsp21.7, their distance between conserved ACD domains was the lowest (0.00). The only GmHsp11.1 in OFM showed typical Cpn10 superfamily characteristics (NCBI–Conserved domains accession: cl09113), with two roof hairpins, 13 putative oligomerisation interfaces, and one mobile loop (Fig 2).

### Phylogenetic analysis and alignment of GmHsps

**Fig 3:**
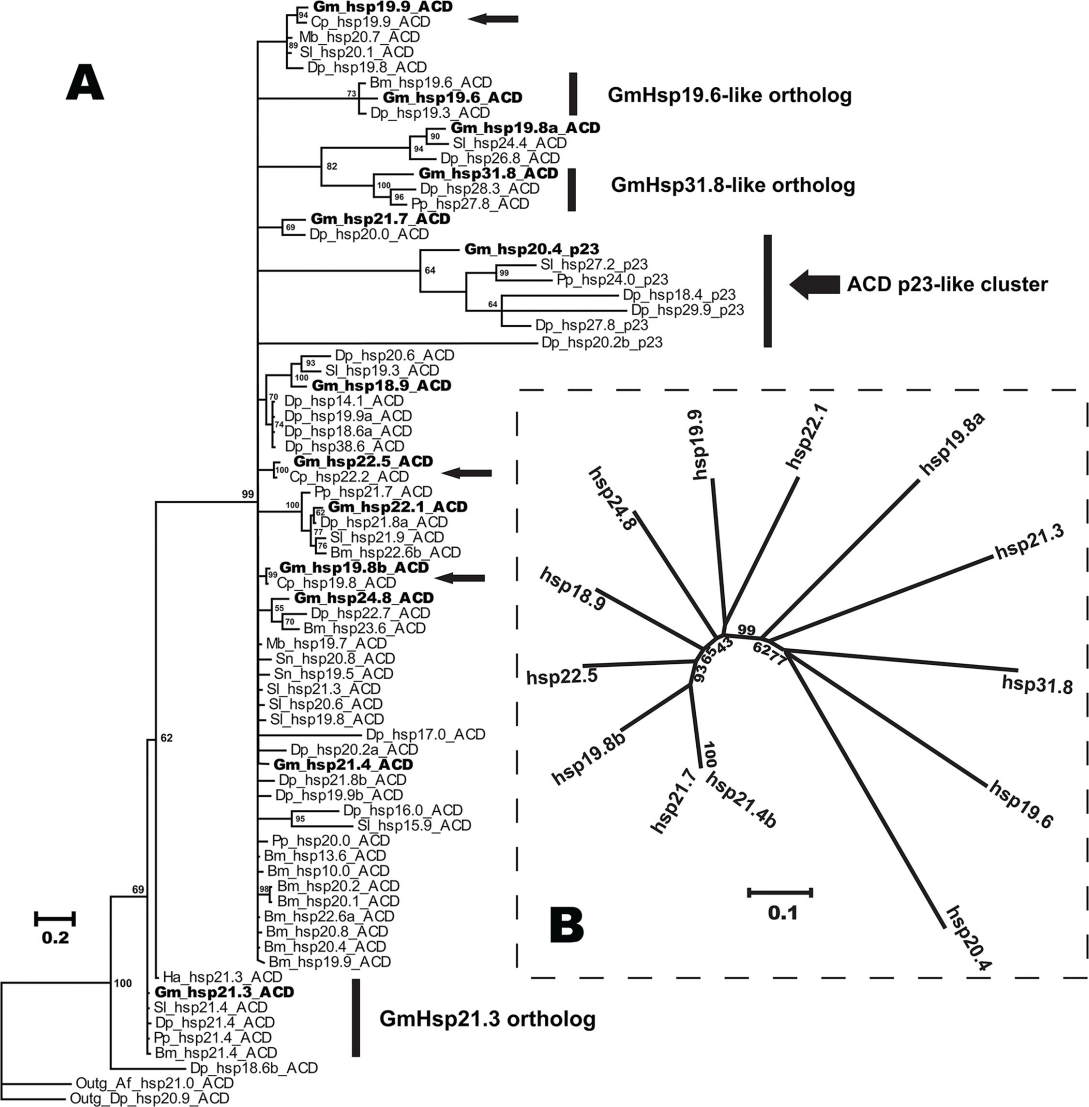
Phylogenetic trees of OFM sHsps. A. Phylogenetic tree of the lepidopteran sHsps. A total of 71 lepidopteran sHsps and two outgroup sequences were used in this Bayesian phylogenetic tree. Only the conserved a-crystalline domains were aligned and used for tree construction. Percentage bootstrap values above 50% were indicated on each cluster. B. An unrooted neighbor-joining tree was constructed with 13 sHsp amino acid sequences from *G*. *molesta*. Bootstrap supports of > 40% are shown on the nodes of the tree. The *G*. *molesta* sHsps are labeled in bold. Thin arrows indicated the close relationship between codling moth and OFM.

To analyze the relationships of OFM sHsps to those of other insects, 71 lepidopteran sHsps including the 13 GmsHsps were collected, and about 100 bp of ACD were truncated to construct Bayesian phylogenetic trees (Fig 3A), which were similar with other trees obtained by different approaches (S1 Fig). The sHsps from lepidopteran species presented two clusters, one with a metazoan ACD and the other one with an ACD p23-like domain, though only with 50% and 73% supporting values respectively. The high level of variation among metazoan ACDs was also evident among lepidopteran sHsps. Three GmHsps showed a close relationship with those from the related species *Cydia pomonella*, from which only three sHsps have been identified so far [17]. The phylogenetic tree also showed that not all the sHsps from the same species clustered in the same branch, as in the case of *Bombyx mori* and *Danaus plexippus*. The orthologous clusters were prevalent in lepidopteran sHsps, including GmHsp19.6-like, GmHsp21.3-like, and GmHsp31.8-like clusters. An unrooted neighbor-joining tree of 13 sHsps was constructed to show the relationship within the sHsp family in this species (Fig 3B). The conserved domain (ACD) of sHsps was clustered with high supporting values, while GmHsp20.4 containing the p23-like domain was isolated. This analysis also indicated that the sHsps with a similar molecular weight did not follow phylogeny, suggesting that the molecular weights of sHsps depended on the variable N- and C-terminal extensions rather than overall similarity.

**Fig 4:**
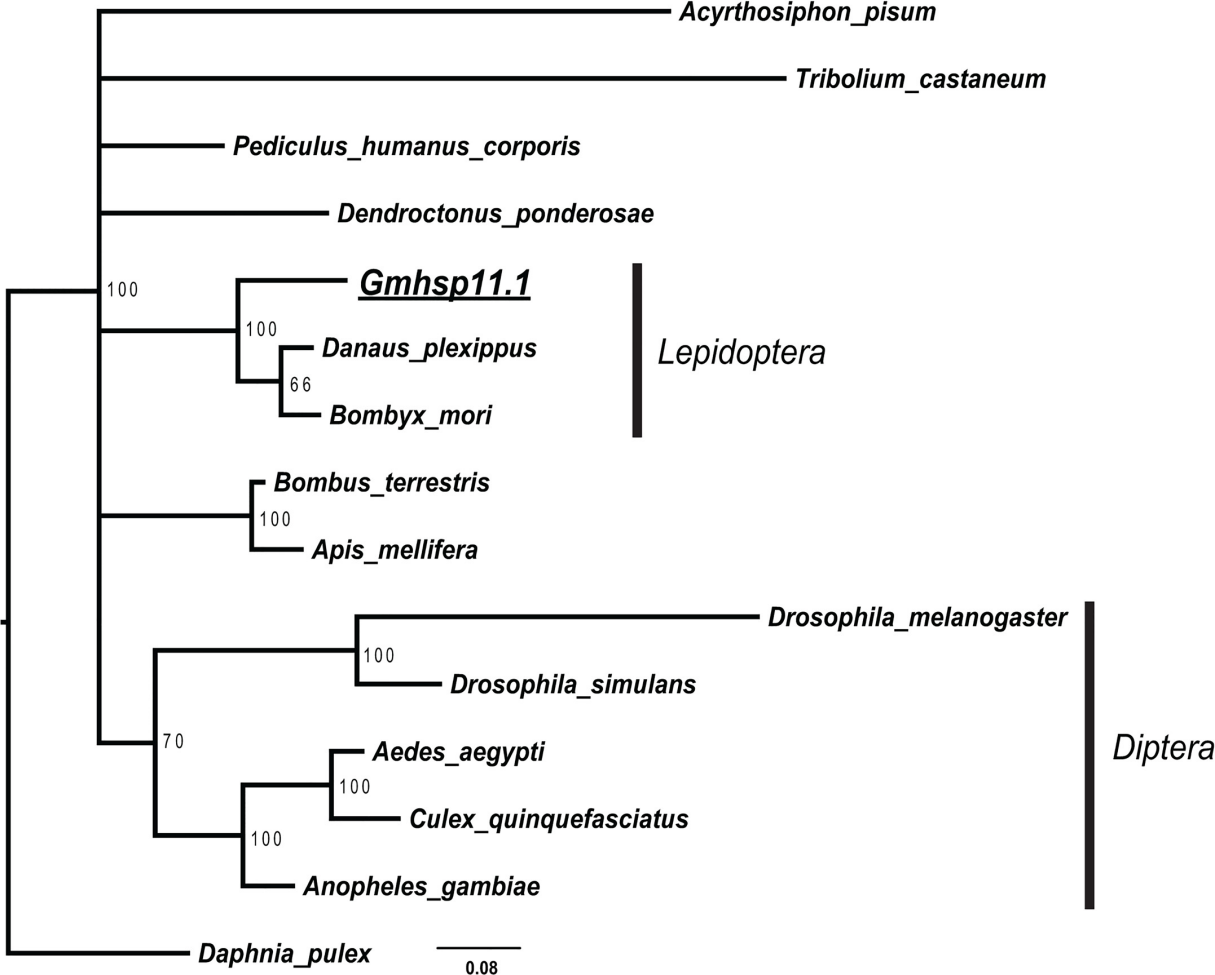
Phylogeny of Cpn10 amino acid sequences of *G*. *molesta* and 14 other insect species. The tree was constructed using the Bayesian method. Bootstrap support above 50% is indicated.

Compared with the complex relationship and high level of divergence of OFM sHsps, GmHsp11.1 of the Cpn10 family exhibited a high level of conservation among insects from different orders (Fig 4). Three lepidopteran Cpn10 genes were clustered within a branch with 100% support. Within the Diptera, the suborders Nematocera (mosquitos) and Brachycera (fruit flies) were separated.

### Expression in response to thermal treatments and diapause

**Fig 5:**
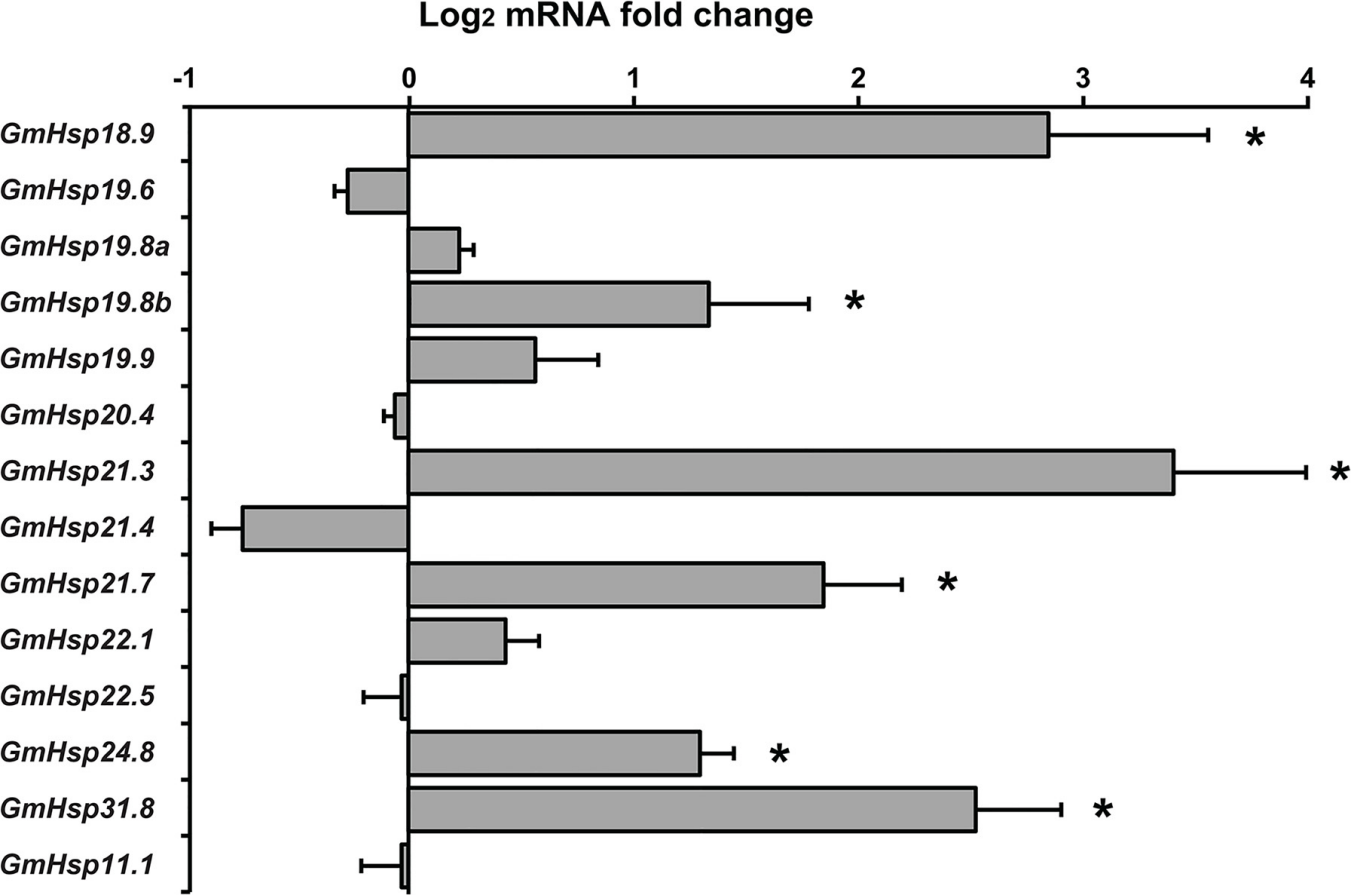
Mean normalized expression (with standard errors) of fourteen GmHsps in diapausing *G*. *molesta*. The values were based on log2 transformation of RT-qPCR of the assayed Hsps. Asterisks indicate mean values that are significantly (P < 0.01) different in 5^th^ instar larvae between diapausing and control groups. A value of 0 indicates no difference in expression, whereas positive and negative values indicate upregulation and downregulation respectively.

Expression of most GmHsp genes was significantly upregulated under thermal stress, with average fold changes relative to controls ranging from 5-fold to 270-fold. In response to heat and cold stress, eight genes (GmHsp19.6, 19.8b, 19.9, 21.4, 21.7, 24.8, 31.8, 11.1) were consistently upregulated, particularly GmHsp19.6, whose expression significantly increased with treatment duration (Tables 1 and 2). Ten GmHsp genes were upregulated under heat shock compared with eight genes under the cold treatment. Three genes (GmHsp19.8a, GmHsp20.4 and GmHsp21.3) did not change in expression during the thermal treatments. The hierarchical cluster analysis for all differentially expressed genes revealed changing patterns of expression for most genes in response to heat and cold stress (S2 Fig). Expression changes of 14 GmHsps in diapausing and nondiapausing OFM are shown in Fig 5. GmHsp 21.3 exhibited the highest fold change in diapause individuals which was highly significant (t = -35.74, df = 8, *p* < 0.001), and together with five other genes showed upregulated expression during diapause.

**Table 1:** Comparison of the overall expression of GmHsp genes under control conditions, after exposure to heat for one hour (H1h), or two hours (H2h) followed by a two-hour recovery period in each case.

Genes	F_(2, 14)_	P	Control	H1h	H2h
GmHsp18.9	16.07	<0.001	a	b	b
GmHsp19.6	67.84	<0.001	a	b	c
GmHsp19.8a	3.84	0.130	a	a	a
GmHsp19.8b	52.4	<0.001	a	b	b
GmHsp19.9	124.82	<0.001	a	b	b
GmHsp20.4	1.78	0.210	a	a	a
GmHsp21.3	1.43	0.356	a	a	a
GmHsp21.4	8.38	0.005	a	ab	b
GmHsp21.7	12.59	0.002	a	b	c
GmHsp22.1	2.38	0.198	a	a	a
GmHsp22.5	38.3	<0.001	a	a	b
GmHsp24.8	50.85	<0.001	a	b	b
GmHsp31.8	17.52	<0.001	a	a	b
GmHsp11.1	105.12	<0.001	a	c	b

Different letters in the same line indicate significant pairwise differences between treatments by Turkey B tests after ANOVA (α = 0.01).

**Table 2:** Comparison of the overall expression of GmHsp genes under control conditions, after exposure to cold for one hour (C1h) or two hours (C2h) followed by two-hour recovery period in each case.

Gene	F_(2, 14)_	P	Control	C1h	C2h
GmHsp18.9	0.501	0.618	a	a	a
GmHsp19.6	51.06	<0.001	a	b	c
GmHsp19.8a	2.53	0.121	a	a	a
GmHsp19.8b	16.3	<0.001	a	ab	b
GmHsp19.9	37.02	<0.001	a	b	b
GmHsp20.4	3.194	0.077	a	a	a
GmHsp21.3	2.69	0.451	a	a	a
GmHsp21.4	9.53	0.003	a	ab	b
GmHsp21.7	21.54	<0.001	a	b	b
GmHsp22.1	2.58	0.117	a	a	a
GmHsp22.5	3.36	0.069	a	a	a
GmHsp24.8	15.76	<0.001	a	b	b
GmHsp31.8	10.13	0.003	a	b	b
GmHsp11.1	18.55	<0.001	a	b	b

Different letters in the same line indicate a significant pairwise difference between treatments by Turkey B tests after ANOVA (α = 0.01).

All GmHsps were detected in each dissected tissue and developmental stage but expressed at different levels (S3 and S4 Figs). GmHsp19.9 and GmHsp21.3 exhibited higher expression levels in the head, with a 4.26 and 4.27 fold change in expression respectively. GmHsp31.8 was the only gene with relatively higher (2.71-fold) expression in the midgut. GmHsp11.1 exhibited high expression during development except at the final larval instar stage and in pupae. GmHsp21.3 showed a 24-fold increase at the 5^th^ instar stage as development proceeded. In contrast, GmHsp20.4 and GmHsp22.1 showed relatively low expression (1-fold and 2-fold on average respectively) across all developmental stages.

### Low phylogenetic signal in lepidopteran sHsp expression patterns

The lepidopteran sHsp genes were tested for phylogenetic signal of expression patterns in Mesquite (S3 Table). Expression responses to the three stresses of heat, cold, and diapause showed no evidence of being conserved within phylogenetic lineages (S5 Fig). This was based on the number of steps expected for each character when compared to the number of steps expected from randomly generated trees. A character was considered to possess phylogenetic signal if there were fewer steps than in 99% of the randomly generated trees. There were four evolutionary steps between two traits in the heat response character, falling out of the range of the null model (mean step = 8.4, range 6–9). For expression changes under cold and diapause conditions there were 15 and 14 evolutionary steps respectively, which did not differ significantly from the 18.1 (range 15–21) and 13.1 (range 11–14) steps predicted under a null model. These findings suggest that there was no detectable phylogenetic signature in functional patterns of the sHsps based on the criteria given.

## Discussion

In the present study, fourteen new members from the sHsp and Hsp10 families have been identified from OFM. The expression levels of nearly half of these sHsp genes changed significantly in response to thermal stresses. These sHsp are likely to be important Hsp members involved in development and stress tolerance in OFM. However, no phylogenetic signal was found in sHsp responses to stress or diapause, suggesting an inconsistency between similarities in sequence, structure and function of the lepidopteran sHsps. This may reflect rapid evolution of sHsp genes towards new functions under various environments.

### High variation of sHsp in sequence feature

All thirteen sHsps in OFM can be separated into two different branches, ACD and p23-like sHsps (Figs 1 and 3). Among them, 12 sHsps were found to contain characteristic ACD, which shows the same number of paralogues of typical ACD in the *D*. *melanogaster* genome [32]. One orthologous group was reported in Lepidoptera [13], and GmHsp21.3 is a member of this common ortholog in OFM. It is very different from sHsps of humans in which all 10 orthologous sHsps can be identified in other mammals [33]. Indeed, most of the sHSPs of Lepidoptera show extensive sequence variation because of the N-terminal arm of divergent sequence and variable length and a C-terminal extension [34,35]. This high variation of sHsp sequences contrasts markedly to conserved patterns for the other Hsp families, including Hsp70, Hsp90, and Hsp10 [36]. GmHsp11.1 was identified as a member of Hsp10 which is highly conserved among insects. The conservation of Hsp10 is as high as for Hsp90 (41–76% vs. 25–88%) [37,38].

### The potential functions of sHsp in temporal and spatial expression

Gene functions are often deduced from known gene families with similar conserved sequence structure [39,40]. Four out of 12 sHsp members of *Drosophila* have been well-characterized for their expression patterns and functions [41], but this information cannot necessarily be used to deduce function for sHsp genes in other insects. The function of Hsp21.3 also has not yet been established even though it is commonly found in Lepidoptera. We thus inferred the potential functions of sHsps in OFM from temporal and spatial patterns of expression in our study and from previous research suggesting that various sHsp may be involved in insect development as well as stress responses. A developmental function for Hsps has been suggested by tissue-specific and tightly regulated patterns of expression during development in *Drosophila* [42–44]. The expression profiles of GmHsp11.1 may point to a putative function in reproduction, embryo development and maternal effects, because of its high expression at the adult and egg stage, in contrast to a steep decrease at the late larval stage. Hps10 is known as a chaperone in mitochondria where it is involved with cell proliferation and differentiation [45]. The increased sHsps (GmHsp18.9, GmHsp19.8a, and GmHsp31.8) from the muscles/cuticle of the abdomen might link to metamorphosis.

GmHsp21.3 may also play a role in development given its increased expression from the neo-larval to pupal stages. The 5^th^ instar stage is key for OFM entering pupal metamorphosis or diapause. High expression of three GmHsp genes (GmHsp19.9, 21.3, and 21.7) in the head of 5^th^ instars may point to a possible function in signal reception and transmission. Many genes are potentially involved with diapause [46,47], and in our study GmHsp21.3 is the most markedly upregulated together with five other GmHsps during early diapause. Hsp23 has previously been shown upregulated in diapausing flesh fly [7,8], while ArHsp21 was shown to be upregulated in diapausing embryos of *Artemia franciscana* [48]. The upregulation of sHsp genes during diapause of OFM may point to a protection function in cold environments, contributing to cold-hardiness of overwintering insects in combination with other Hsps such as Hsp70 [49]. However, there was no common feature of sHsps that were upregulated in diapausing insects.

Our expression results suggest that sHsps may be important in sub-lethal temperature tolerance in OFM, acting as molecular chaperones under extreme conditions. In OFM, sub-lethal temperatures frequently arise in different seasons. For example, low temperatures at night often occur in early spring or late autumn when OFM adults might regulate expression of genes such as GmHsp19.6 and GmHsp21.7. Hot days in summer that are increasing in frequency under climate warming might induce expression of genes like GmHsp19.9 and GmHsp24.8, particularly in adults that cannot easily find a refuge unlike larvae inside plant tissue.

### Lack of phylogenetic signal of sHsps expression for function divergence

We analyzed sequence and expression data available for sHsps to test for potential functional conservation (heat, cold and diapause) among lepidopteran insects. We found that similarities in sequence were inconsistent with functional conservation in sHsp genes, as assessed by the association between stress responses and the expression of sHsps across a lepidopteran phylogeny covering five families. Our findings do not fit in well with the notion that conserved domains can be used to extrapolate gene function from model species to target species under investigation. This may reflect the fact that sHsps with the unified structural ACD do not display conserved functional binding sites affecting expression patterns. Those sHsps with relatively constant expression patterns may have “housekeeping” roles in maintaining molecular structures at certain stages [50] rather than acting as conventional stress chaperones.

The inconsistent sHsp response patterns suggest that sHsp responses are evolutionarily labile. While sHsps are induced at high temperatures in many organisms [51], not all described sHsps show increased expression when organisms are placed under temperature stress [12,14,52]. Given the large number of sHsps in organisms, it is possible that only a subset is required for chaperone functions to maintain cellular viability. This may leave sHsps free to evolve additional functions apart from needing to act as stress chaperons and protective agents [42]. The absence of strong phylogenetic signal points to functional constraints being restricted to closely related groups rather than higher taxonomic levels, even though sHsps might not necessarily always diverge after speciation, as in the case of the ortholog hsp21.3.

Genome sequence data is continuing to expand knowledge about the structure of sHSPs [34,35], but the most sHsp families in insects remain poorly characterized. Thus, more information is needed to further assess the presence/absence of phylogenetic signal and links to functional roles. This involves testing more species with divergent traits (particularly for cold and diapause responses) and also characterizing expression patterns in more detail. Signal might be present but not evident from whole organism comparisons; for instance lepidopteran species might exhibit temporal and/or spatial variation in expression. In addition, the classification of sHsp by molecular weight and conserved domains should be further characterized, and functional tests need to also focus on manipulations of gene expression such as through RNAi [53].

## Supporting Information

S1 Fig Phylogenetic trees of OFM sHsps based on the conserved a-crystalline domains (about 105 amino acids) for the tree construction. A. The NJ tree with bootstrap support > 40% on the nodes. B. The ML tree with bootstrap support > 40% on the nodes. The *G*. *molesta* sHsps are labeled in bold. Thin arrows indicated the close relationship between codling moth and OFM.
(DOCX)

S2 Fig Hierarchical clustering of differentially expressed genes in response to heat and cold treatments (1h and 2h, respectively). (DOCX)

S3 Fig Normalized mRNA expression levels of the fourteen GmHsps in different tissues. Error bars are standard errors.
(DOCX)

S4 Fig Normalized mRNA expression of the 14 GmHsp genes in OFM at different stages. Error bars are standard errors.
(DOCX)

S5 Fig Phylogenetic tree of the lepidopteran species considered, including the state of change in the branches and tips in three characters involving heat (A), cold (B), and diapause (C) responses. (DOCX)

S1 Table The GmsHsp real-time PCR primers, annealing temperatures, and expected fragment lengths. (DOCX)

S2 Table List of sHsps of Lepidopteran species used in phylogenetic analysis. (DOCX)

S3 Table List of sHsp expression information used in lepidopteran phylogenetic signal detection. (DOCX)
