# Supplementary material for: Stress Responses of Small Heat Shock Protein Genes in Lepidoptera Point to Limited Conservation of Function across Phylogeny
Source: PLoS One. 2015 Jul 21;10(7):e0132700. doi: 10.1371/journal.pone.0132700 (PMC4511463; doi:10.1371/journal.pone.0132700)
Supplement: S1 Fig — A. The NJ tree with bootstrap support > 40% on the nodes. B. The ML tree with bootstrap support > 40% on the nodes. The G. molesta sHsps are labeled in bold. Thin arrows indicated the close relationship between codling moth and OFM. (DOCX) [file pone.0132700.s001.docx]

**S3.** **Phylogenetic trees of OFM sHsps based on the conserved a-crystalline domains for the tree construction.** A. The NJ tree with bootstrap support proportions > 40% on the nodes. B. The ML tree with bootstrap support proportions > 40% on the nodes. The *G. molesta* sHsps are labeled in bold. Thin arrows indicated the close relationship between codling moth and OFM.

**A.**





**B.**

**
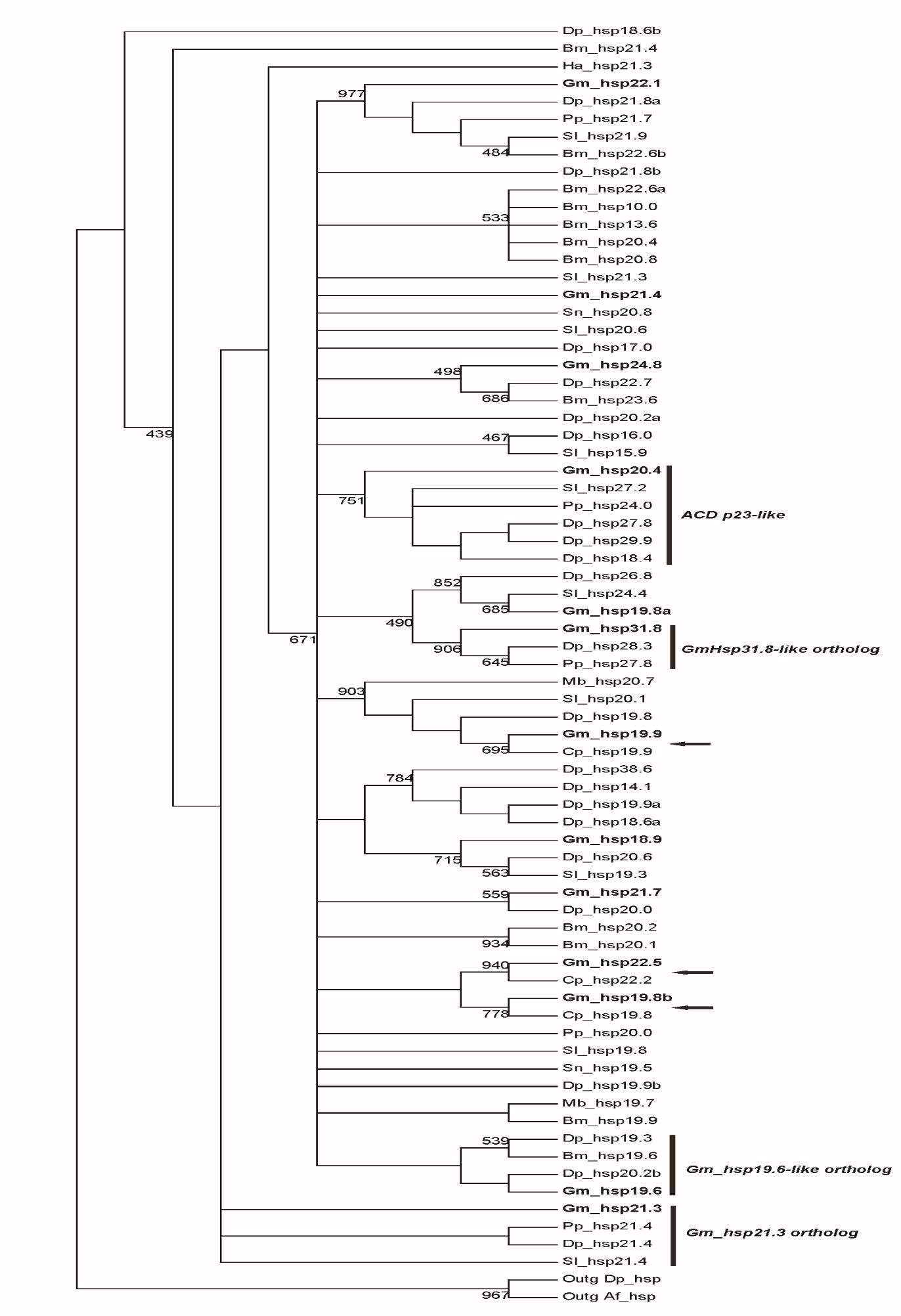
**
