# Supplementary material for: Stress Responses of Small Heat Shock Protein Genes in Lepidoptera Point to Limited Conservation of Function across Phylogeny
Source: PLoS One. 2015 Jul 21;10(7):e0132700. doi: 10.1371/journal.pone.0132700 (PMC4511463; doi:10.1371/journal.pone.0132700)

**S4. Hierarchical clustering of differentially expressed genes in response to heat and cold treatments (1h and 2h, respectively).** A complete linkage algorithm was used to cluster fourteen differentially expressed sHsp genes. Horizontal stripes represent genes and columns show experimental treatments. Logarithmic fold change of treatment vs. control are shown in the heat map using red and green color codes for up- and down-regulation, respectively.


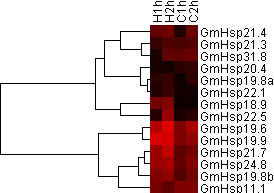

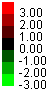

Supplement: S2 Fig — (DOCX) [file pone.0132700.s002.docx]
