# Supplementary material for: Stress Responses of Small Heat Shock Protein Genes in Lepidoptera Point to Limited Conservation of Function across Phylogeny
Source: PLoS One. 2015 Jul 21;10(7):e0132700. doi: 10.1371/journal.pone.0132700 (PMC4511463; doi:10.1371/journal.pone.0132700)

**S6. Normalized mRNA expression of the 14 GmHsp genes in OFM different stages. Error bars are standard errors.**


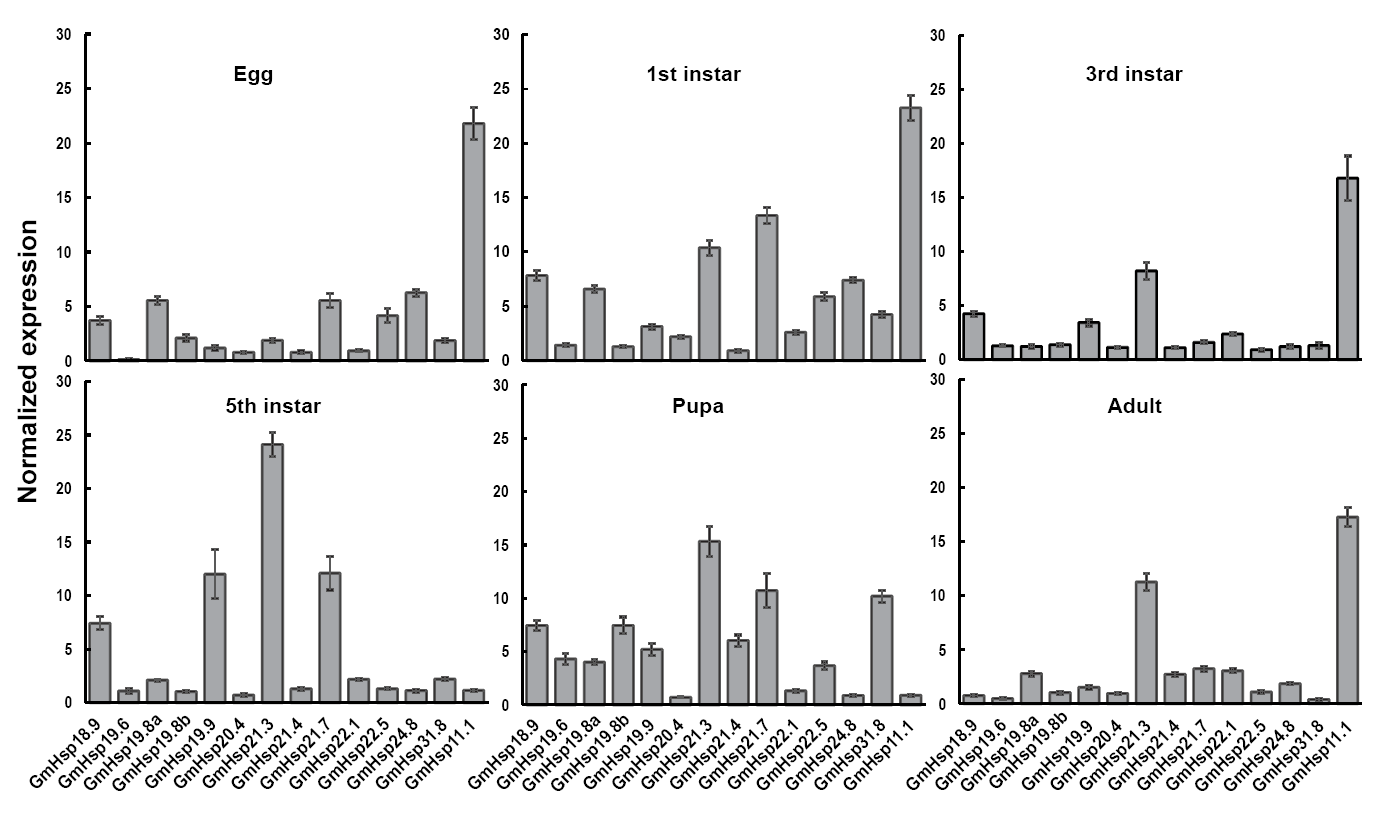

Supplement: S4 Fig — Error bars are standard errors. (DOCX) [file pone.0132700.s004.docx]
