# Supplementary material for: Stress Responses of Small Heat Shock Protein Genes in Lepidoptera Point to Limited Conservation of Function across Phylogeny
Source: PLoS One. 2015 Jul 21;10(7):e0132700. doi: 10.1371/journal.pone.0132700 (PMC4511463; doi:10.1371/journal.pone.0132700)

**S8 Fig. Phylogenetic tree of the lepidopteran species considered, including the state of change in the branches and tips in three characters involving heat (A), cold (B), and diapause (C) responses.**


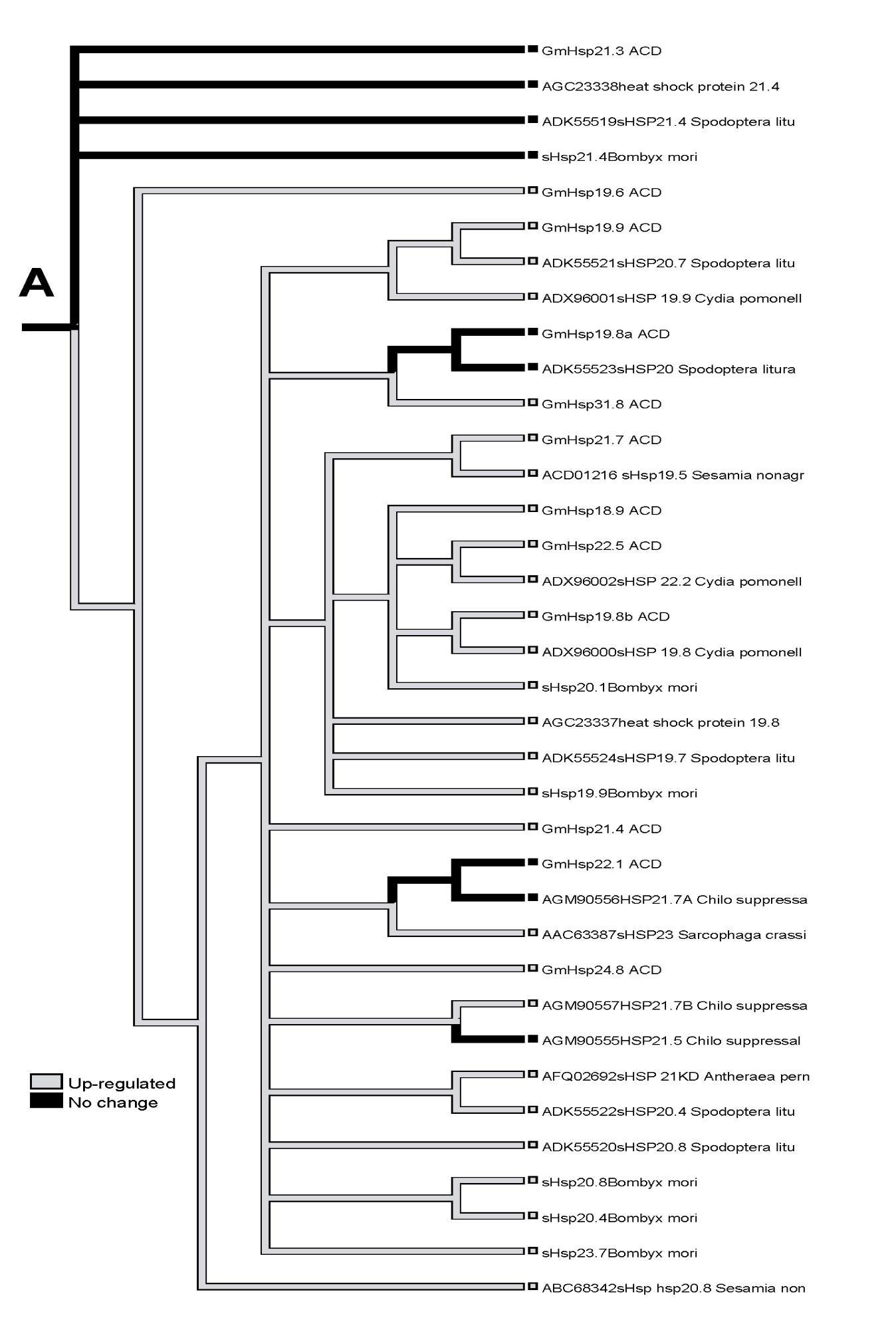


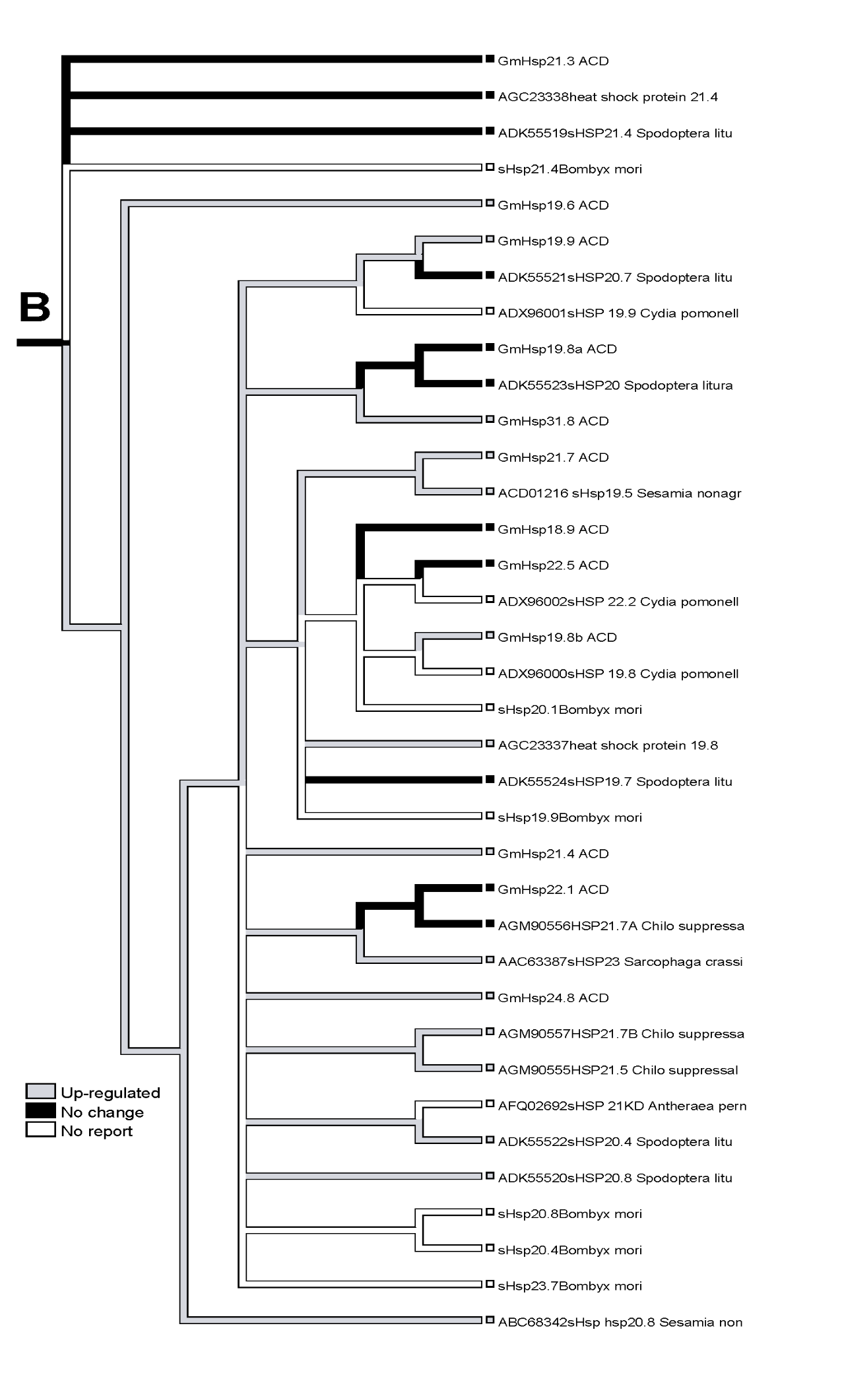


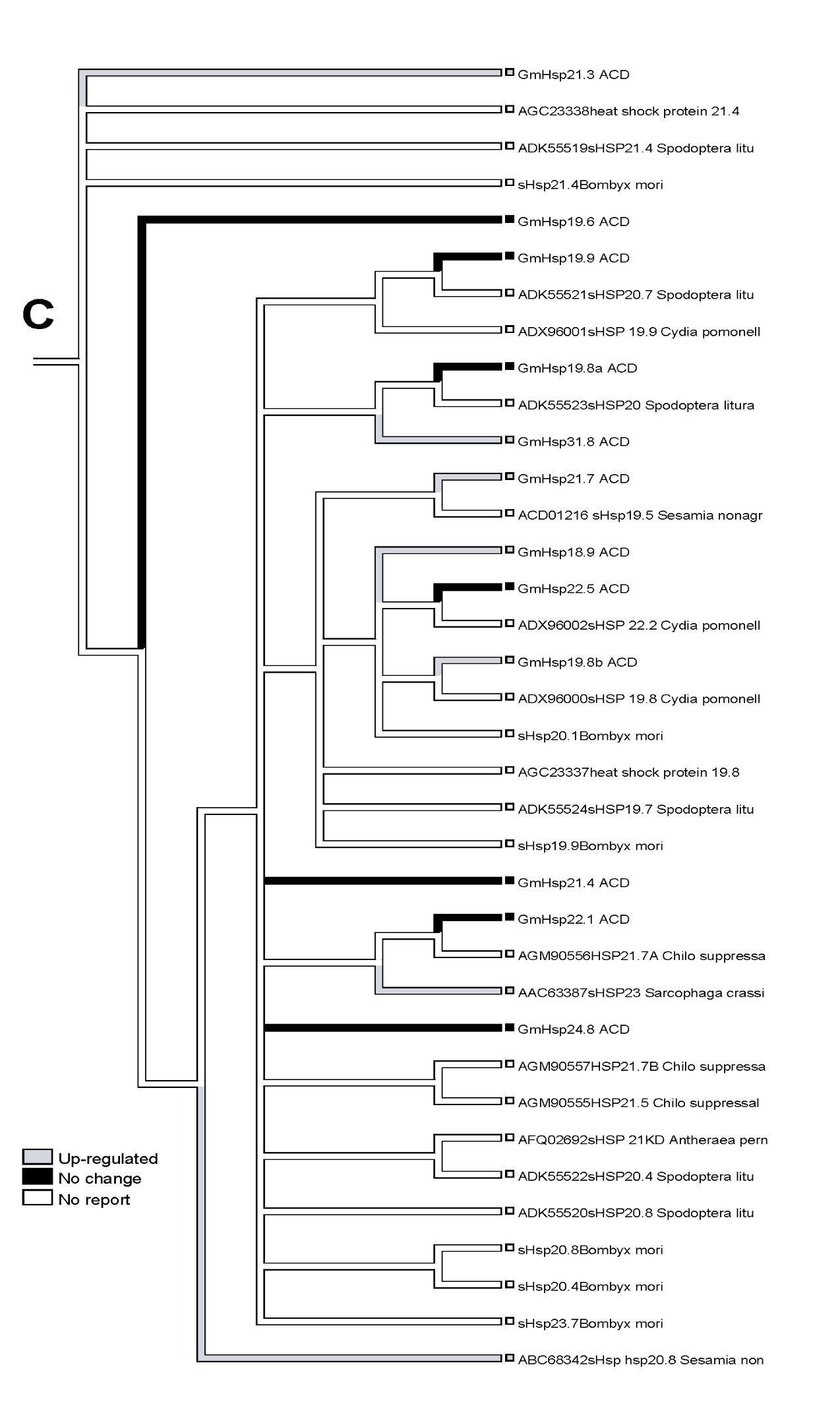

Supplement: S5 Fig — (DOCX) [file pone.0132700.s005.docx]
