# Supplementary material for: Stress Responses of Small Heat Shock Protein Genes in Lepidoptera Point to Limited Conservation of Function across Phylogeny
Source: PLoS One. 2015 Jul 21;10(7):e0132700. doi: 10.1371/journal.pone.0132700 (PMC4511463; doi:10.1371/journal.pone.0132700)
Supplement: S1 Table — (DOCX) [file pone.0132700.s006.docx]

**S1. The GmsHsp real-time PCR primers, annealing temperatures, and expected fragment lengths.**

| **GENES** | **PRIMER (F/R)** | **Tm** | **SIZE (bp)** |
| --- | --- | --- | --- |
| GmHsp21.3 | ACGAGGAGAAGTCGGACAC | 56 | 165 |
|  | GGATGCTTCGGTCGGTGAT |  |  |
| GmHsp21.4 | CATCGGTTCCAGCATCAAAG | 56 | 153 |
|  | ATGTACCCGTGGTCGTCT |  |  |
| GmHsp19.6 | TCCTTTCGCTGTATTCACTC | 58 | 149 |
|  | CTAATTGCACCTCGACTTTC |  |  |
| GmHspp19.9 | GAAGAAAGGCAAGACGAGC | 56 | 141 |
|  | CGCATTCAAAGGAGCAGTA |  |  |
| GmHsp19.8a | TTTGACCGTAAATGCTCCG | 56 | 138 |
|  | GCTGATGGTGGTGTAGTTG |  |  |
| GmHsp21.7 | GAGGACATAAGCGTCAAAGT | 58 | 141 |
|  | CACGGTATCAGGCAAGCAG |  |  |
| GmHsp20.4 | AATGATAACACGGAACAGGA | 56 | 144 |
|  | CACAGGCACAAATTCTACATC |  |  |
| GmHsp31.8 | GCTCGCAAGCACCATCATAAA | 56 | 139 |
|  | GGTTGTCCTCGGCGTCCAT |  |  |
| GmHsp18.9 | GGCTTTCGTCTGATGGTGT | 56 | 140 |
|  | CTAAACTTCCTTCTGTGGCTC |  |  |
| GmHsp22.5 | AACGGAGATACACTTTGCCT | 58 | 157 |
|  | TGCTTCCTCCTAACTGGTC |  |  |
| GmHsp22.1 | CCTTGTCTTCCGATGGTGT | 56 | 160 |
|  | ACGATGGGTACGGTCTGTT |  |  |
| GmHsp19.8b | ATGGAACAGGACTTTGGGCT | 56 | 147 |
|  | CTTATCGGACTTGATGGTGG |  |  |
| GmHsp24.8 | GACAGTTCGTAAGGAGGTA | 53 | 135 |
|  | CCACTCTTTCCGATTTGTTT |  |  |
| GmHsp11.1 | GTCATCGCTGTGGGACCTG | 58 | 132 |
|  | CTCCTTGTCTTCGGAATCTAA |  |  |
| Gm_GAPDH | GGAAAGCTGACTGGTATGG | 56 | 167 |
|  | ACCTGGTCCTCGGTGTAG |  |  |
| Gm_Actin | CAAGGAGCCTCAGTTTATCA | 56 | 98 |
|  | CCTCAACCGCTTTCGTCAT |  |  |
