# Supplementary material for: Stress Responses of Small Heat Shock Protein Genes in Lepidoptera Point to Limited Conservation of Function across Phylogeny
Source: PLoS One. 2015 Jul 21;10(7):e0132700. doi: 10.1371/journal.pone.0132700 (PMC4511463; doi:10.1371/journal.pone.0132700)
Supplement: S2 Table — (DOCX) [file pone.0132700.s007.docx]

**S2. List of sHsps of lepidopteran species used in phylogenetic analysis.**

| Species | Name | # Amino Acids | Weight_Da | GenBank # | NEW NAME | TREE LABEL |
| --- | --- | --- | --- | --- | --- | --- |
| *Bombyx_mori* | hsp20.1 | 178 | 20152.63 | ACM24346 | hsp20.2 | Bm_hsp20.2_ACD |
|  | hsp20.1 | 178 | 20138.6 | NP_001036941 | hsp20.1 | Bm_hsp20.1_ACD |
|  | hsp20.1 | 199 | 22630.7 | ACM24354 | hsp22.6a | Bm_hsp22.6a_ACD |
|  | hsp20.8 | 186 | 20804.46 | NP_001091794 | hsp20.8 | Bm_hsp20.8_ACD |
|  | hsp20.8 | 181 | 20427.07 | NP_001037038 | hsp20.4 | Bm_hsp20.4_ACD |
|  | hsp19.9 | 177 | 19890.48 | NP_001036984 | hsp19.9 | Bm_hsp19.9_ACD |
|  | hsp23.7 | 209 | 23570.75 | NP_001036942 | hsp23.6 | Bm_hsp23.6_ACD |
|  | hsp23.7 | 198 | 22561.5 | NP_001091767 | hsp22.6b | Bm_hsp22.6b_ACD |
|  | hsp19.5 | 167 | 19554.3 | NP_001164470 | hsp19.6 | Bm_hsp19.6_ACD |
|  | hsp19.5 | 122 | 13551.16 | AAK06407 | hsp13.6 | Bm_hsp13.6_ACD |
|  | hsp21.4 | 187 | 21403.99 | NP_001036985 | hsp21.4 | Bm_hsp21.4_ACD |
|  | NA | 90 | 9956.18 | AAK06409 | hsp10.0 | Bm_hsp10.0_ACD |
| *Danaus_plexippus* | HSP25.4 | 255 | 29855.2 | EHJ63088 | hsp29.9 | Dp_hsp29.9_p23 |
|  | HSP20.4 | 149 | 17002.8 | EHJ63492 | hsp17.0 | Dp_hsp17.0_ACD |
|  | HSP20.4 | 177 | 20603.1 | EHJ63493 | hsp20.6 | Dp_hsp20.6_ACD |
|  | hsp19.8 | 167 | 18644.9 | EHJ63499 | hsp18.6a | Dp_hsp18.6a_ACD |
|  | HSP20.4 | 181 | 20209.7 | EHJ63989 | hsp20.2a | Dp_hsp20.2a_ACD |
|  | hsp19.8 | 352 | 38586.9 | EHJ67066 | hsp38.6 | Dp_hsp38.6_ACD |
|  | hsp19.5 | 165 | 19311.1 | EHJ67172 | hsp19.3 | Dp_hsp19.3_ACD |
|  | hsp19.8 | 178 | 19865.4 | EHJ68318 | hsp19.9a | Dp_hsp19.9a_ACD |
|  | hsp1 | 191 | 21800.7 | EHJ68903 | hsp21.8a | Dp_hsp21.8a_ACD |
|  | hsp19.8 | 129 | 14121.7 | EHJ69639 | hsp14.1 | Dp_hsp14.1_ACD |
|  | hsp21.4 | 187 | 21389.9 | EHJ69746 | hsp21.4 | Dp_hsp21.4_ACD |
|  | hsp23.7 | 198 | 22682.7 | EHJ70499 | hsp22.7 | Dp_hsp22.7_ACD |
|  | HspB1 | 166 | 18602.9 | EHJ71411 | hsp18.6b | Dp_hsp18.6b_ACD |
|  | hsp25.4 | 242 | 27813.9 | EHJ73481 | hsp27.8 | Dp_hsp27.8_p23 |
|  | NA | 179 | 20210.5 | EHJ74218 | hsp20.2b | Dp_hsp20.2b_p23 |
|  | hsp19.7 | 189 | 21825.7 | EHJ74663 | hsp21.8b | Dp_hsp21.8b_ACD |
|  | hsp33.6 | 248 | 28343.2 | EHJ77259 | hsp28.3 | Dp_hsp28.3_ACD |
|  | NA | 243 | 26798.7 | EHJ77261 | hsp26.8 | Dp_hsp26.8_ACD |
|  | NA | 177 | 20004.9 | EHJ77276 | hsp20.0 | Dp_hsp20.0_ACD |
|  | NA | 174 | 19797.3 | EHJ77277 | hsp19.8 | Dp_hsp19.8_ACD |
|  | hsp19.7 | 177 | 19890.5 | EHJ77540 | hsp19.9b | Dp_hsp19.9b_ACD |
|  | hsp19.8 | 137 | 15964.2 | EHJ77787 | hsp16.0 | Dp_hsp16.0_ACD |
|  | hsp25.4 | 159 | 18418.5 | EHJ78247 | hsp18.4 | Dp_hsp18.4_p23 |
| *Cydia_pomonella* | hsp19.8 | 177 | 19768.3 | ADX96000 | hsp19.8 | Cp_hsp19.8_ACD |
|  | hsp19.9 | 175 | 19910.5 | ADX96001 | hsp19.9 | Cp_hsp19.9_ACD |
|  | hsp22.2 | 192 | 22172.8 | ADX96002 | hsp22.2 | Cp_hsp22.2_ACD |
| *Mamestra_brassicae* | hsp20.7 | 180 | 20700.3 | BAF03557 | hsp20.7 | Mb_hsp20.7_ACD |
|  | hsp19.7 | 175 | 19673.3 | BAF03558 | hsp19.7 | Mb_hsp19.7_ACD |
| *Sesamia_nonagrioides* | hsp20.8 | 185 | 20828.5 | ABC68342 | hsp20.8 | Sn_hsp20.8_ACD |
|  | hsp19.5 | 174 | 19512.1 | ACD01216 | hsp19.5 | Sn_hsp19.5_ACD |
| *Papilio_polytes* | NA | 191 | 21728.6 | BAM18936 | hsp21.7 | Pp_hsp21.7_ACD |
|  | hsp20 | 187 | 21389.9 | BAM19038 | hsp21.4 | Pp_hsp21.4_ACD |
|  | NA | 248 | 27826.7 | BAM19083 | hsp27.8 | Pp_hsp27.8_ACD |
|  | NA | 212 | 24013.5 | BAM19146 | hsp24.0 | Pp_hsp24.0_p23 |
|  | NA | 178 | 19980.6 | BAM19244 | hsp20.0 | Pp_hsp20.0_ACD |
| *Helicoverpa_armigera* | hsp21.4 | 187 | 21345.9 | AGC39039 | hsp21.3 | Ha_hsp21.3_ACD |
| *Spodoptera_litura* | NA | 187 | 21375.9 | ADK55519 | hsp21.4 | Sl_hsp21.4_ACD |
|  | NA | 189 | 21341.1 | ADK55520 | hsp21.3 | Sl_hsp21.3_ACD |
|  | NA | 176 | 20118.6 | ADK55521 | hsp20.1 | Sl_hsp20.1_ACD |
|  | NA | 182 | 20576.2 | ADK55522 | hsp20.6 | Sl_hsp20.6_ACD |
|  | NA | 221 | 24402.5 | ADK55523 | hsp24.4 | Sl_hsp24.4_ACD |
|  | NA | 175 | 19792.4 | ADK55524 | hsp19.8 | Sl_hsp19.8_ACD |
|  | hsp15.9 | 138 | 15919.4 | AFK14098 | hsp15.9 | Sl_hsp15.9_ACD |
|  | hsp19.3 | 171 | 19317.9 | AFK14099 | hsp19.3 | Sl_hsp19.3_ACD |
|  | hsp22.0 | 193 | 21949.7 | AFK14100 | hsp21.9 | Sl_hsp21.9_ACD |
|  | hsp27.2 | 243 | 27202.7 | AFK14101 | hsp27.2 | Sl_hsp27.2_p23 |
| OUTGROUP |  |  |  |  |  |  |
| *Artemia_franciscana* | hsp21 | 181 | 21048 | ABD19712 | hsp21.0 | Outg_Af_hsp21.0_ACD |
| *Daphnia_pulex* | hsp20 | 180 | 20941.1 | EFX75568 | hsp20.9 | Outg_Dp_hsp20.9_ACD |
