# Supplementary material for: Stress Responses of Small Heat Shock Protein Genes in Lepidoptera Point to Limited Conservation of Function across Phylogeny
Source: PLoS One. 2015 Jul 21;10(7):e0132700. doi: 10.1371/journal.pone.0132700 (PMC4511463; doi:10.1371/journal.pone.0132700)
Supplement: S3 Table — (DOCX) [file pone.0132700.s008.docx]

**S7. List of sHsp expression information used in lepidopteran phylogenetic signal detection.**

| **Gene** | **GenBank #** | **Reference** |
| --- | --- | --- |
| Sesamia nonagrioides_Hsp19.5 | ACD01216 | 窗体顶端  Gkouvitsas et al., 2008窗体底端 |
| Sesamia nonagrioides_Hsp20.8 | ABC68342 | Gkouvitsas et al., 2008 |
| Chilo suppressalis_Hsp19.8 | AGC23337 | Lu et al., 2014 |
| Chilo suppressalis_Hsp21.4 | AGC23338 | Lu et al., 2014 |
| Chilo suppressalis_Hsp21.7a | AGM90556 | Lu et al., 2014 |
| Chilo suppressalis_Hsp21.7b | AGM90557 | Lu et al., 2014 |
| Chilo suppressalis_Hsp21.5 | AGM90555 | Lu et al., 2014 |
| Antheraea pernyi_Hsp21 | AFQ02692 | Lin et al., 2013 |
| Spodoptera_litura_Hsp21.4 | ADK55519 | Shen et al., 2011 |
| Spodoptera_litura_Hsp20.8 | ADK55520 | Shen et al., 2011 |
| Spodoptera_litura_Hsp20.7 | ADK55521 | Shen et al., 2011 |
| Spodoptera_litura_Hsp20.4 | ADK55522 | Shen et al., 2011 |
| Spodoptera_litura_Hsp20 | ADK55523 | Shen et al., 2011 |
| Spodoptera_litura_Hsp19.7 | ADK55524 | Shen et al., 2011 |
| Cydia_pomonella_Hsp19.8 | HQ219475 | Garczynski et al., 2011 |
| Cydia_pomonella_Hsp19.9 | HQ219476 | Garczynski et al., 2011 |
| Cydia_pomonella_Hsp22.2 | HQ219477 | Garczynski et al., 2011 |
| Bombyx_mori_Hsp20.1 | AB195971 | Sakano et al., 2006 |
| Bombyx_mori_Hsp20.8 | AF315317 | Sakano et al., 2006 |
| Bombyx_mori_Hsp20.4 | AF315318 | Sakano et al., 2006 |
| Bombyx_mori_Hsp19.9 | AB195970 | Sakano et al., 2006 |
| Bombyx_mori_Hsp23.7 | AB195973 | Sakano et al., 2006 |
| Bombyx_mori_Hsp21.4 | AB195972 | Sakano et al., 2006 |
| Gm_hsp19.6 |  | Our study |
| Gm_hsp19.9 |  | Our study |
| Gm_hsp19.8a |  | Our study |
| Gm_hsp21.7 |  | Our study |
| Gm_hsp21.3 |  | Our study |
| Gm_hsp20.4 |  | Our study |
| Gm_hsp31.8 |  | Our study |
| Gm_hsp21.4 |  | Our study |
| Gm_hsp18.9 |  | Our study |
| Gm_hsp22.5 |  | Our study |
| Gm_hsp22.1 |  | Our study |
| Gm_hsp19.8b |  | Our study |
| Gm_hsp24.8 |  | Our study |
